# Default Positions: How Neuroscience’s Historical Legacy has Hampered Investigation of the Resting Mind

**DOI:** 10.3389/fpsyg.2012.00321

**Published:** 2012-09-10

**Authors:** Felicity Callard, Jonathan Smallwood, Daniel S. Margulies

**Affiliations:** ^1^Centre for Medical Humanities, Durham UniversityDurham, UK; ^2^Department of Social Neuroscience, Max Planck Institute for Human Cognitive and Brain SciencesLeipzig, Germany; ^3^Max Planck Research Group Neuroanatomy and Connectivity, Max Planck Institute for Human Cognitive and Brain SciencesLeipzig, Germany

**Keywords:** resting state, daydreaming, default-mode network, mind-wandering, history of cognitive neuroscience

## Abstract

The puzzle of the brain and mind at rest – their so-called default state – is strongly influenced by the historical precedents that led to its emergence as a scientific question. What eventually became the default-mode network (DMN) was inaugurated via meta-analysis to explain the observation that the baseline “at rest” condition was concealing a pattern of neural activations in anterior and posterior midline brain regions that were not commonly seen in external-task-driven experiments. One reason why these activations have puzzled scientists is because psychology and cognitive neuroscience have historically been focused on paradigms built around external tasks, and so lacked the scientific and theoretical tools to interpret the cognitive functions of the DMN. This externally-focused bias led to the erroneous assumption that the DMN is the primary neural system active at rest, as well as the assumption that this network serves non-goal-directed functions. Although cognitive neuroscience now embraces the need to decode the meaning of self-generated neural activity, a more deliberate and comprehensive framework will be needed before the puzzle of the wandering mind can be laid to rest.

## A Spontaneous-thought Experiment

Imagine a society in which people are preoccupied with shape and color – which is to say that a person, when left to her own devices, spends all her so-called “idle time” reflecting on all manner of different colors and forms. Suppose that such thoughts could be interrupted by momentary cognitive demands, such as a standard visual paradigm or word-generation task provided by an experimenter. In this imaginary society, the “default mode” – meaning the brain state during the baseline condition – would engage brain regions involved in reflecting on shapes and colors, and would have a well-defined neural basis because of the systematic cognitive activity required to reflect on visual imagery.

Now consider that researchers in this imaginary society are fascinated by the study of self-related thinking (core components of proposed default-mode function in our own society) and have developed multiple experimental designs through which to investigate it. In such a context, brain activity observed during the so-called “baseline” state would reflect society’s collective obsession with shape and color, but might seem hard to interpret for researchers preoccupied with dissecting the various components of self-related thinking^1^. Even though, outside the lab, scientists might freely admit to spending much of their lives thinking about chromatic and geometric variation, it might well take a surprising length of time for even thoughtful researchers to realize that they have relatively few psychological theories available to understand this peculiar “baseline” state – a state during which they had acquired a significant amount of data.

Our hypothetical example illustrates the situation facing today’s cognitive neuroscientists as they struggle to understand what we might call the dark side of cognition – or to use Marcus Raichle’s formulation, the mental functions accompanying “the brain’s dark energy” (Raichle, 2006). While neuroscientists have long embraced the need to decode the processes and functions underlying spontaneous neural activity (for a comprehensive overview, see Buzsáki, 2006), cognitive neuroscience – as well as cognitive psychology, the discipline to which it is much indebted – is hampered in this regard by its historical legacy. Scientists are at a point at which, to analogize through use of the hypothetical scenario above, they are beginning to realize that their expertise in investigating and modeling self-related thinking might not seamlessly translate into paradigms that will allow them to grasp chromatic and geometric variation. Moreover, as our hypothetical scenario makes clear, the “default mode” of mental states ought not to be conceptualized as a purely biological phenomenon. These mental states, after all, exist within a cultural context: the operations of an unconstrained mind will undoubtedly be shaped by the form and values of the culture within which they unfold. In this article, we consider how the historical context in which the default-mode network (DMN) was documented influenced explanations of the functions that the system played. We additionally demonstrate how the complex phenomena that comprise “self-generated thought” have been misinterpreted because of neuroscience’s failure to acknowledge how its preoccupation with goal-focused external processing has skewed its assumptions about the operations of the mind during so-called “idle” time.

It is necessary to emphasize that “self-generated thought” is an umbrella term that incorporates – and often conflates – several concepts at the edges of cognitive neuroscience. Task-unrelated thoughts (Fransson, 2006), “spontaneous cognition” (Andrews-Hanna et al., 2010b), “mind-wandering” (Smallwood and Schooler, 2006; Mason et al., 2007), “free association” (Andreasen et al., 1995; Freed, 2008), “self-focused attention” (Gentili et al., 2009), or “introspectively oriented” thought (Fransson, 2005) are commonly used as synonyms for “self-generated thought.” Such terms should not, we argue, necessarily be regarded as equivalent; nor should it be assumed that an individual’s mental processes, when she is left undisturbed, are encapsulated by one singular construct or phenomenological experience. While we are mindful of these complexities, we use the overarching term “self-generated thought” throughout the current article, as it emphasizes the opposition to “generated in response to perception” (Smallwood, in press). This conceptual contrast is important given that perceptually guided thought has been the framework that has been the focus of much research in cognitive psychology and cognitive neuroscience; indeed, it is the consequences of this dyadic framework for the current state of knowledge as regards the mind at rest with which we are preoccupied in this article.

## Experimental Tasks: Psychology’s Default Mode

The difficulties faced by neuroscientists in their attempts to investigate self-generated cognition (which became the conceptual basis for DMN function) have their roots in the twentieth-century experimental techniques that were applied to understand the mind. If one pauses and introspects upon one’s own mental life, it becomes evident that states of self-generated mental activity are not just common, but are central to the phenomenological experience of being human. Although introspection reinforces the conclusion that self-generated mental processes are important, skepticism regarding the validity of such a technique relegated investigations of self-generated cognition to the margins of research for almost half a century.

Watson (1913) in his behaviorist manifesto argued that “introspection forms no essential part of [psychology’s] methods” and that psychology “need no longer delude itself into thinking that it is making mental states the object of observation.” To develop psychology as a practical science, behaviorism jettisoned the study of consciousness as the object of study, and hence the introspective techniques promoted by scientists such as Wundt and Titchener (see Danziger, 1980) gave way to the use of external stimuli to explore both human and animal behavior. In the late 1950s the cognitive revolution resurrected the importance of internal states in understanding human behavior, and yet psychology continued to employ a task-based approach. Psychologists largely relied on paradigms that used an external probe, and were more comfortable with interpreting objective (behavioral) responses (such as button-presses) than subjective responses (in the form of the subject’s accounts of her experiential states in relation to the probes; Jack and Roepstorff, 2002).

The progression of the twentieth century increased the array of dependent measures available to psychologists, as did the categories of psychological life that were studied. While studies of patients with specified brain lesions (e.g., Scoville and Milner, 1957) provided the foundations of a brain-based explanation for the mind, the advent of modern neuroimaging techniques (e.g., PET and fMRI) enabled researchers to measure brain activity during covert mental processes while participants performed psychological tasks. The 1980s witnessed creative collaborations between neuroscientists and cognitive psychologists, whose ability to dissect human behavior using experimental task-based designs provided a basis for cognitive neuroscience.

At this point, the discipline was a neural extension of the reaction-time model (which the nineteenth-century physiologist, Donders, 1969, had used), in which a mental process is isolated by subtracting a control state from a task state (Raichle, 1998). Brain activity cannot be measured in absolute terms and so to localize brain areas that are more active during a specific task, a neutral, so-called “baseline” brain state is needed from which increases can be identified. Task-based neuroimaging studies tended to use a resting baseline (often a fixation cross), which was assumed to be a neutral comparison to task-induced brain activity. However, researchers began to notice that sometimes during this control condition a constellation of regions, often on the medial surface of both hemispheres, exhibited heightened activation relative to external tasks (Buckner, 2012). These serendipitous findings emerged in the context of a paradigm that was not expecting them – and that did not initially have the conceptual tools with which adequately to interpret them (see Callard and Margulies, 2011 for an historical analysis of the complex emergence of the new scientific object that would become the “DMN”). In 2001, Marcus Raichle and colleagues published three papers (Gusnard and Raichle, 2001; Gusnard et al., 2001; Raichle et al., 2001) that described the baseline resting state as the “default mode” of function, and argued that because the observed decreases in brain activity appeared to be “largely task independent,” there might “be an organized mode of brain function” that is present as a baseline or default state and is suspended during specific goal-directed behaviors (Gusnard et al., 2001, p. 4259). Two years later, Greicius et al. (2003) coined the functional-anatomical term “DMN,” arguing that it “account[ed], in large part, for the phenomenon of task-related decreases in brain activity.” Here, it is important to bear in mind that the DMN refers to a specific set of neural regions, whereas the *default mode* is used to describe the state underlying self-generated cognitions that emerge when individuals are left idle. Although the network has become the manifest functional correlate of the default mode, these two basic concepts should not be equated on a one-to-one basis.

## The Influence of Historical Context on Conceptions of the Default-Mode Network

There are at least two ways in which early formulations of the DMN were hampered by the historical context in which they emerged. First, because neuroscience had an almost exclusive focus on external-task-based experiments during the 1990s, the majority of studies included in the first meta-analysis (Shulman et al., 1997) showing “task-induced deactivations” required the continuous focus of attention and motor responses, as well as a capacity to concentrate for reasonable durations. As the DMN was defined by neural regions that were not involved in the experiments included in the meta-analysis, its uniqueness during the resting state was at least in part a historical quirk of the experiments conducted over the prior decade: if research had been dominated by experiments probing different psychological constructs, our capacity to interpret the activity of the DMN – as well as what was in fact delineated *as* the DMN – would likely have been different. Indeed, it is important to emphasize that the degree to which the DMN – and, indeed, other networks, appear to be “activated” during “at rest” states will inevitably depend on the nature of the experimental task condition to which the resting state is compared. (If, for example, the task condition centrally engages autobiographical memory and prospective thinking, then there may well appear to be very little activation of the DMN during the “at rest” periods.)

It is now evident that the DMN should not be conflated with the activity of the mind *in toto* at resting state. Smith et al. (2009) found that the same networks activated by tasks (as derived from a meta-analysis) were also correlated in activity during the resting state. These findings demonstrate that the DMN is in no way commensurate with spontaneous neural activity, as it is now clear that the whole brain exhibits functionally organized behavior when not occupied by an external task. This suggests that spontaneous neural activity should not be seen as a hallmark of the DMN, and so raises deep questions about how to determine which elements of spontaneous neural activity relate to which forms of conscious, self-generated thought. The relationship between “functional correlations” and “functional activity,” as well as the relationship between both of these phenomena and conscious thought are undoubtedly conceptually complex, as well as very difficult to investigate experimentally using fMRI^2^. The extent to which cognitive neuroscience as a whole has grasped the scale of these experimental as well as conceptual challenges is far from clear.

Neuroscientists have faced obstacles in their efforts to identify and disentangle these elements in part because the historical bias toward explicating external processing has meant that the psychological vocabulary for describing internally generated mental content is relatively stunted. But it should be emphasized that from the late nineteenth century onward, both psychology and related disciplines (e.g., psychiatry and psychoanalysis) show evidence of fascinating, though largely marginalized investigations into states of mind that depart from the well-studied construct of externally focused attention. James (1890), in his *Principles of Psychology*, for example, reflected on that “curious state of inhibition” that tends to be produced by “[f]atigue” and “monotonous mechanical occupations that end by being automatically carried on.” Indeed, James’s definition of attention explicitly encompasses both internal and external trains of thought – an aspect of attention that was largely neglected for many decades. Moreover, there is a compelling body of literature – largely unknown or disregarded in cognitive psychology – that documents various methods to investigate the processes characterizing activities pursued during so-called “idle time” (e.g., Green, 1923; Singer, 1966; Antrobus, 1968). These include introspective methods, the free association methods of psychoanalysis, visualization, and guided imagery methods. Some use (boring) external stimulation tasks precisely to provoke unconstrained mental activity, while others attempt to capture such activity more directly. This literature demonstrates a complicated lineage of overlapping terms for what we now refer to as self-generated mental activity – including daydreaming, fantasy, mind-wandering, and dissociation. It is not difficult to see that if these research domains had flourished within cognitive psychology rather than been neglected, our vocabulary for describing self-generated thought would have been richer; our methods to investigate such thought more creative; and hence our capacities to interpret the psychological meaning of different forms of spontaneous neural activity in different systems, including the mental life and associated neural systems of the DMN, more substantial.

The second way that historical pressures influenced interpretations of DMN functions also arose from the observation that such systems exhibited activity in conditions under which the neural systems engaged in task-based experiments were absent. Researchers concluded from these observations that the psychological processes that these regions serve are non-goal directed: Raichle and colleagues’ initial papers on the default function, for example, assumed that “spontaneous … mental activity” (which was described as stimulus-independent thoughts, daydreams, task-unrelated imagery, free association, or stream of consciousness) occurred when “subjects are not actively engaged in goal-directed behavior” (Gusnard and Raichle, 2001). This line of argumentation resulted in the network itself being described in 2005 as *task-negative* (Fox et al., 2005; Fransson, 2006). Further cementing this view that the networks served dichotomous functions was the observation that a network of dorsolateral prefrontal and superior parietal brain regions were found to be “anti-correlated” with activity within the DMN at rest (Fox et al., 2005). This counterbalanced interaction between such large-scale networks was considered by several publications to have cognitive significance (Hampson et al., 2006; Kelly et al., 2008), and to be a core aspect of functional brain organization. As such, this apparent “see-sawing” of neural activity between two widespread brain networks suggested rather intuitive – and folk-psychological – distinctions between opposing psychological functions of goal-oriented cognition and spontaneous thought. Thus the second way that the historical context influenced our notions of the DMN arose from cognitive science’s conflation of goal-directed thought with external processing.

Recent evidence has begun to challenge the core assumption of the task-negative view of DMN function by demonstrating that the internally generated thought that this system supports can have a goal-driven component. For example, when task demands are minimal, self-generated thought occurs most frequently in those individuals who perform well on goal-driven tasks (Levinson et al., 2012); while self-generated thought can be associated with a reduction in the processing of distracter stimuli, a hallmark process by which individuals stay on task to achieve goals (Barron et al., 2011). Finally, self-generated thought is generally focused on the future (Andrews-Hanna et al., 2010a; D’Argembeau et al., 2010; Stawarczyk et al., 2011), suggesting the experience serves the function of autobiographical planning (Baird et al., 2011; Smallwood and O’Connor, 2011; Smallwood et al., 2011). Similarly, evidence from cognitive neuroscience demonstrated that periods of self-generated thought can engage both elements of the dorsolateral prefrontal cortex and the DMN (Christoff et al., 2009), and under these circumstances connectivity between these regions is higher than when on task (Christoff, 2012). In addition, the experience of mind-wandering and the process of autobiographical planning entail activity in both the DMN and the executive system (Spreng et al., 2010; Gerlach et al., 2011).

Self-generated thought and the DMN can, therefore, perform goal-directed functions, and when doing so engage broadly similar domain general cognitive and neural processes as when external-goal-related tasks are performed. The initial formulation of the DMN as a task-negative network engaged in “idle” mental processes was in part a legacy of the historical tendency to study hidden mental processes through the lens of external, perceptually driven tasks. Critically, self-generated activity arises from memory (rather than perception), and thus external tasks can provide only limited insight into the psychological features of these forms of cognition.

A good example of how understanding self-generated thought through the lens of an external task can lead to erroneous conclusions can be seen in studies linking the experience to error. Research in the first decade of the twenty-first century on daydreaming and mind-wandering has demonstrated that self-generated material (gathered through self-report methods) could be associated with worse external attention (for a review see Smallwood and Schooler, 2006). In neuroscience, the activity of the DMN demonstrated a similar property: its activity was heightened in the period prior to poor external task performance (Weissman et al., 2006). At a general level, the observation that self-generated thought is a barrier to external processing is hardly surprising: perhaps the most well documented fact about brain function is that it cannot perform an infinitely large number of tasks simultaneously. Nonetheless, these observations served two critical functions. First, they clarified that self-generated material had an observable consequence on data that could be verified at the third-person level, a necessary requirement for its scientific analysis. Second, they helped to provide a working hypothesis regarding what should be explained: these studies could show that the experience of self-generated thought is intermittent and so entails (i) a process by which attention ebbs and flows between an internal and external focus and (ii) a system with a limited capacity that cannot attend to everything at the same time.

These data were, however, often used to make an additional inference: that the occurrence of self-generated states during task performance indicated an absent-minded or unintended lapse in the capacity to focus attention on the task (McVay and Kane, 2010). While it is undoubtedly correct that difficulties in control can lead to problems focusing on external information, this does not mean that all examples of self-generated thought result from failures to control attention, *even* if they are detrimental to a task. As individuals can choose to engage in self-generated thought, the only conclusion that can be drawn from studies linking self-generated thought, or DMN activity, to poor external attention is that the architecture of the brain means that external and internal processes are in competition for limited resources (Smallwood, 2010). Again, we see how the strong disciplinary preoccupation with externally directed attention influenced the assumptions used to interpret the characteristics of self-generated thought.

## Moving Beyond Default Positions

We have demonstrated that disciplinary legacies and preoccupations have shaped neuroscientists’ conceptions of the default mode, the DMN, and self-generated thought in non-trivial ways. In order to move beyond the default positions that have tended to be advanced in relation to these phenomena, it is important that neuroscientists and their cognitive psychology collaborators first of all acknowledge some of the significant limitations of these default positions. Such acknowledgment would, in turn, open the possibility of more extensive engagement with marginalized literature (from the past – and increasingly the present) that offers conceptual and methodological creativity in exploring these phenomena. The growing fascination with resting-state functional connectivity fMRI within cognitive neuroscience will not, in and of itself, necessarily result in the field becoming better at exploring and understanding the contents of consciousness that individuals self-generate when at rest. To make progress on this front, it will be necessary to integrate characterizations of phenomenological experiences with measurement approaches that extend beyond contrast-based methodologies of traditional fMRI.

In addition, it is worth reflecting with some seriousness on the implications of the thought experiment with which we introduced our article. Studies have documented that the DMN is engaged in self-referential autobiographical thought (Mitchell et al., 2006), but the extent to which the DMN supports a relatively *domain general* psychological process, rather than reflects a system that is tied to a *specific* form of content, is far from clear. Just as the preoccupations of a scientific community can fluctuate over time, the social and experimental context in which the research subject is embedded will surely influence the unconstrained thoughts that she engages in when given the opportunity to do so. Consider a comparison of the default modes of scientists who are preoccupied with shapes, color and other forms of visual imagery with those who excel at playing chess or at attuning themselves to their own and others’ emotions. Would these individuals’ minds be similar or different when at rest? While the content of unconstrained thought is likely to be affected by the values of the society and/or communities to which those individuals belong, the general lack of interest from neuroscientists in earlier research that has attempted to explore such questions (e.g., studies in Singer, 1966) means that we are far from being able adequately to answer this question. It is time to recognize that the intricacies of self-generated thought cannot be understood simply by the application of standard neural cognitive approaches: to move beyond the default positions that neuroscience adopts when studying self-generated thought, it will be necessary to adopt specific theories and methods that are tailored to understanding the different qualities of experiences that fall within this category.

## Conflict of Interest Statement

The authors declare that the research was conducted in the absence of any commercial or financial relationships that could be construed as a potential conflict of interest.

## Footnotes

^1^We are not implying that there is no overlap between brain activations associated with self-related thinking, and those with processing shape and color.

^2^We thank one of the reviewers for this important point.
